# Leptin attenuates cerebral ischemic injury in rats by modulating the mitochondrial electron transport chain via the mitochondrial STAT3 pathway

**DOI:** 10.1002/brb3.1200

**Published:** 2019-01-10

**Authors:** Shijun Hu, Daobin Cheng, Dingtian Peng, Jing Tan, Yanlan Huang, Chunyong Chen

**Affiliations:** ^1^ Department of Neurology Hainan General Hospital Haikou China; ^2^ Department of Neurology The First Affiliated Hospital of Guangxi Medical University Nanning China

**Keywords:** cerebral ischemia, leptin, mitochondria, neuroprotection, STAT3

## Abstract

**Background:**

According to recent studies, leptin may exert a neuroprotective function by affecting the phosphorylation of signal transducer and activator of transcription 3 (STAT3). During stress, STAT3 regulates mitochondrial oxidative stress and reduces apoptosis.

**Objective:**

In the present study, we hypothesized that leptin increases STAT3 phosphorylation in the mitochondria and protects against mitochondrial oxidative stress in rats subjected to permanent middle cerebral artery occlusion (MCAO).

**Results:**

Leptin reduced reactive oxygen species (ROS) production, and we confirmed that the mechanism underlying this change involved the enzymatic activities of mitochondrial respiratory chain complexes I and II. In addition, leptin increased the level of STAT3 Ser727 phosphorylation in the mitochondria.

**Conclusions:**

Based on these results, leptin may regulate mitochondrial respiratory chain enzymatic activities via mitochondria‐targeted STAT3 to reduce ROS production and protect brain tissues from mitochondrial oxidative stress during cerebral ischemia.

## 1 INTRODUCTION

Acute ischemic cerebrovascular disease is the most common cerebrovascular disease and is a common cause of death and disability. According to several recent studies (Davis, Mudd, & Hawkins, 2014; Mancini et al., 2014; Valerio et al., 2009), leptin represents a potential therapy for ischemic cerebrovascular disease, providing another possibility for treating stroke.

Leptin, which is primarily secreted by adipocytes, is encoded by the obese gene (Halaas et al., 1995). The neuroprotective effect of leptin was recently shown to be closely associated with the phosphorylation of signal transducer and activator of transcription 3 (STAT3) in a middle cerebral artery occlusion (MCAO) model (Amantea et al., 2011). STAT3 has more important physiological functions than other STAT proteins, and it is widely involved in neuron growth, differentiation, and apoptosis, among other processes (Levy & Lee, 2002). During ischemic stress, mitochondrial STAT3 reduces the generation of reactive oxygen species (ROS) by partially blocking electrons that pass through complexes I and II (Szczepanek, Chen, Larner, & Lesnefsky, 2012). Although STAT3 has been reported to play an important role in the neuroprotective effect of leptin (Amantea et al., 2011), little is known about the role of mitochondrial STAT3 in this effect. Thus, in this study, we further investigated whether leptin affects mitochondrial oxidative stress in the ischemic core and penumbral regions through the mitochondrial STAT3 signaling pathway.

## 2 EXPERIMENTAL PROCEDURES

### 2.1 Animal model and drug treatments

Adult male Sprague–Dawley rats (weighing 250–280 g) were supplied by the Experimental Animal Center of Guangxi Medical University. All experimental procedures were reviewed and approved by the Experimental Animal Ethics Committee of Guangxi Medical University and strictly followed during the course of the experiment.

Rats were fasted for 12 hr before surgery but were given free access to drinking water. Ischemia was induced in the rat brains by occluding the middle cerebral artery using the filament occlusion method originally described by Longa and Weinstein (8), with slight modifications. Briefly, the rats were anesthetized with pentobarbital sodium (40 mg/kg, intraperitoneally). Hair was removed from the surgical area, and the area was disinfected. During surgery, the body temperature was monitored using a rectal probe. An electric blanket was used to maintain a body temperature of 37 ± 0.5°C. The rats were fixed in the supine position. The right common carotid artery, external carotid artery, and internal carotid artery were isolated by a midline incision in the neck. An incision was made 4 mm from the carotid artery bifurcation. A silicone‐coated nylon filament (diameter: 0.36 mm, JiaLing Corporation, Guangzhou, Guangdong, China) was inserted through the incision into the right common carotid artery and gently advanced into the internal carotid artery, ~18 mm from the carotid artery bifurcation, until slight resistance was felt. The thread was then tightly fastened with a suture line to prevent bleeding. The incision was closed in layers and disinfected with iodophor. Rats were placed in separate animal cages and injected with sterile saline (20 ml/kg, intraperitoneally) to prevent dehydration. The body temperatures of the rats were maintained as they recovered in their cages. After regaining consciousness, the rats were allowed free access to food and water.

Each animal was randomly assigned to one of three groups: (a) the sham‐operated group (sham group), which received the same surgery but without blocking or ligation of the blood vessel; (b) the cerebral ischemia group (vehicle group), which was administered normal saline 3 hr before MCAO (1 ml/kg via hypodermic injection); and (c) the recombinant rat leptin (PeproTech, Rocky Hill, NJ, USA) injection group (leptin group), which was administered leptin 3 hr before MCAO (1 mg/kg via hypodermic injection).

### 2.2 Evaluation of brain edema and neurological deficits

Six hours after permanent MCAO, the brains were harvested and immediately separated into the left and right hemispheres, cerebellum, and brainstem (*n* = 5 animals per group). The two hemispheres were separated and weighed individually using a precise electronic balance. The extent of brain edema was expressed as follows: brain edema = (damaged ipsilateral hemisphere weight) – (contralateral hemisphere weight) (Zhang et al., 2013). Neurological deficits in the rats were examined at 6 hr after permanent MCAO (*n* = 5 animals per group). The deficits were scored using a modified Longa (Longa, Weinstein, Carlson, & Cummins, 1989) scale as follows: 0 = no symptoms of nerve injury; 1 = paralysis of the front/hind legs, legs cannot be fully extended; 2 = circling toward the contralateral side; 3 = falling toward the contralateral side; 4 = cannot walk spontaneously, loss of consciousness; and 5 = death.

The following exclusion criteria were used (Zhang et al., 2013):

1. Death within 6 hr of MCAO,
2. Longa score of 0 (6 hr after MCAO), and
3. Subarachnoid hemorrhage (assessed macroscopically during brain sampling).

### 2.3 Neuropathology and measurement of local infarct proportion

Six hours after permanent MCAO, the cerebral infarct volume was evaluated using 2,3,5‐triphenyltetrazolium chloride (TTC) staining (*n* = 5 animals per group). Briefly, the rats were anesthetized and the brains were rapidly removed from the skulls. Serial 2‐mm sections were prepared from the anterior to posterior direction. Sections were stained with a solution of 2% TTC in saline at 37°C to measure ischemic damage. After a 15‐min incubation, sections were fixed with a 4% paraformaldehyde solution and stored at 4°C until further analysis.

Images of the TTC‐stained sections were captured using a digital scanner and analyzed using image analysis software (ImageJ, version 1.30, US National Institutes of Health, Bethesda, MD, USA). The data are presented as the percentage of infarct area/ipsilateral hemisphere area in the coronal slices.

### 2.4 Tissue processing for mitochondrial isolation

Regions corresponding to the ischemic core and penumbra from the right (ipsilateral) hemisphere designated for biochemical analysis were dissected using procedures described by Ashwal et al. (1998), with some modifications. The core (striatum and overlying cortex) was separated from the penumbra (adjacent cortex) by creating transverse diagonal cuts at approximately the “2 o'clock” and “5 o'clock” positions (ipsilateral hemisphere), respectively.

The mitochondria were isolated from the cerebral ischemic core and the penumbra by differential centrifugation using a mitochondrial extraction kit (Jiancheng Bioengineering Institute, Nanjing, JS, China). The freshly removed cerebral ischemic core and penumbra tissue samples were weighed and immediately placed in ice‐cold sterile saline to remove impurities. Samples were then completely shredded and grounded in a Dounce glass tissue grinder with lysis buffer. Homogenates were centrifuged at 800× *g* for 10 min. Then, the supernatants were retained, and cell nuclei, large membrane fragments, and intact cells were removed. The supernatants were centrifuged at 15,000× *g* for 10 min, and the precipitates were retained. The precipitates were then resuspended in wash buffer, centrifuged again at 15,000× *g* for 10 min, and the final precipitates were retained. The isolated mitochondria were either cryopreserved in preservative buffer at −70°C or immediately used in experiments.

### 2.5 Western blotting

After the mitochondrial purification process, mitochondrial samples from the core and penumbral regions were mixed with RIPA lysis buffer, phosphatase and protease inhibitors, and phenylmethylsulfonyl fluoride and gently shaken for 15 min at 4°C (*n* = 4 animals per group). The mitochondrial samples were subsequently centrifuged (13,523× *g*, 4°C) for 15 min. The supernatant containing mitochondrial protein extracts was retained, and the concentrations of mitochondrial proteins were determined using the bicinchoninic acid assay method. Each sample was separated using 10%–15% sodium dodecyl sulfate‐polyacrylamide gel electrophoresis and electrotransferred onto polyvinylidene fluoride membranes (Millipore, Chelmsford, MA, USA). Membranes were blocked with 5% nonfat milk powder dissolved in a Tween‐20 phosphate‐buffered saline solution for 2 hr. Then, membranes were washed and incubated with primary antibodies against STAT3 (1:2,000, Cell Signaling Technology) and phospho‐STAT3 (p‐STAT3) (1:1,000, Cell Signaling Technology) overnight at 4°C, followed by an incubation with an IRdye680‐conjugated secondary antibody (1:15,000, LI‐COR Biosciences) for 2 hr. Protein bands were quantified using a LI‐COR Odyssey infrared imager (LI‐COR Biosciences, Inc.).

### 2.6 Measurement of ROS levels

The core and penumbra samples from each group were prepared according to the manufacturer's instructions to determine the total intracellular ROS levels (Jiancheng Bioengineering Institute, Nanjing, JS, China) (*n* = 5 samples per group). Samples were mechanically homogenized in an ice‐water bath and then centrifuged at 1,000 g for 10 min. Supernatants were collected for measurements and were thoroughly mixed with a 10 mM 2,7‐dichlorodihydrofluorescein diacetate solution, followed by an incubation for 30 min at 37°C. The intracellular ROS levels were measured using a spectrophotometer (SHIMADZU, RF‐5301PC).

### 2.7 Measurement of the activities of mitochondrial respiratory chain complexes (MRCCs) I‐IV

The mitochondrial respiratory chain enzymatic activities were analyzed with Mitochondrial Respiratory Chain Complex Activities Assay Kits (Jianchen, Nanjing, China) and quantified using a dual wavelength/single wavelength ultraviolet spectrophotometer (VARIAN CARY 50) (*n* = 4 samples per group). All analyses of enzymatic activities were conducted at 30°C, except for the analysis of complex IV, which was conducted at 25°C. The purified mitochondria were ruptured in an ice‐water bath using ultrasound. The colorimetric assay began within 3 s of the addition of mitochondrial lysate. Complex I (reduced form of nicotinamide‐adenine dinucleotide (NADH)‐coenzyme Q reductase) activity was presented as the μmol of NADH oxidized/min/mg of protein and was measured with or without the complex I inhibitor rotenone by assessing the rate of NADH oxidation at wavelengths of 340 and 380 nm. Complex II (succinate‐coenzyme Q reductase) activity was reported as the μmol of dichlorophenol‐indophenol (DCIP) reduced/min/mg of protein and was measured by assessing the rate of DCIP reduction at 600 nm. Complex III (ubiquinol‐cytochrome c oxidoreductase) activity was reported as the μmol of cytochrome c reduced/min/mg of protein and was measured by assessing the rate of cytochrome c reduction at 550 nm with or without the specific ubiquinol‐cytochrome c reductase inhibitor antimycin A. Complex IV (cytochrome c oxidoreductase) activity was reported as the μmol of cytochrome c oxidized/min/mg of protein and was determined by measuring cytochrome c oxidation at 550 nm.

### 2.8 Statistical analysis

Statistical analyses were conducted using SPSS software (version 17.0) (SPSS, Chicago, IL, USA). The numerical data are presented as means ± standard deviations. The between‐group differences, except for the neurological deficit data, were assessed using one‐way analysis of variance, followed by the least significant difference post hoc test or Student's *t* test. The neurological deficit data were analyzed using the Mann–Whitney *U* test. Differences were considered statistically significant at the 0.05 level.

## 3 RESULTS

### 3.1 Leptin alleviated brain edema and neurological deficits

**Figure 1:**
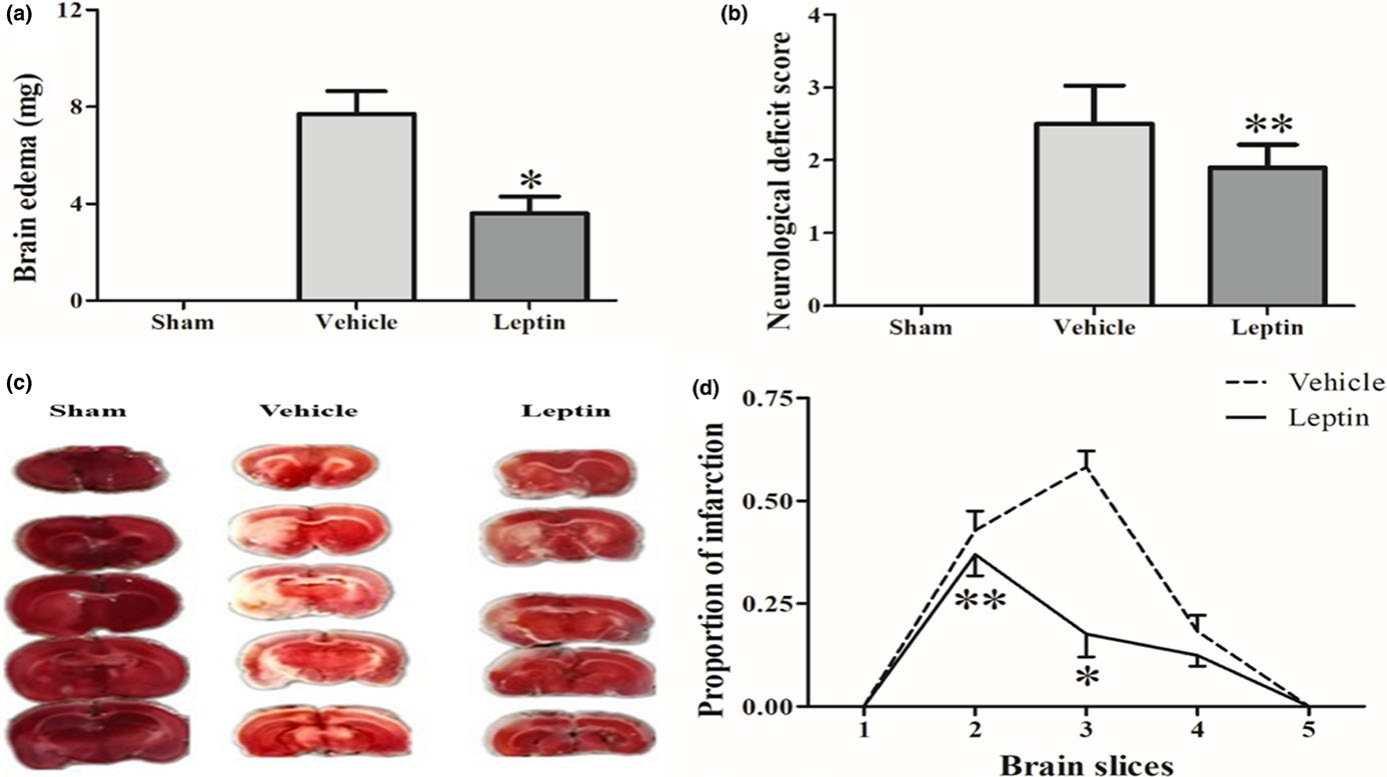
Leptin alleviated brain edema (a), neurological deficits (b), and infarct areas (c and d) at 6 hr after permanent MCAO in rats. Changes in brain edema were determined by calculating the difference between the impaired ipsilateral cerebral hemisphere weight and the contralateral cerebral hemisphere weight (a). A representative photograph of TTC staining (c) and the corresponding proportion of the area exhibiting cerebral infarction at 6 hr after permanent MCAO (d). The proportion of the cerebral infarct area was measured using ImageJ software after TTC staining. ^*^p < 0.001 compared with the vehicle group; ^**^p < 0.05 compared with the vehicle group (*n* = 5 rats per experimental group)

Cerebral ischemic injury is accompanied by varying degrees of edema. The degree of edema significantly differed between the leptin and vehicle groups. At 6 hr after permanent MCAO, the leptin group exhibited a milder degree of brain edema than the vehicle group (53.2% decrease, *p* < 0.001) (Figure 1a).

The neurological deficits were scored using the modified Longa scale. Consistent with previous studies (Amantea et al., 2011; Zhang et al., 2013), our results confirmed that the leptin group presented significantly fewer neurological deficits (*p* < 0.05) than the vehicle group 6 hr after permanent ischemia (Figure 1b).

### 3.2 Leptin reduced the infarct area

We examined the proportion of the infarcted area in brain slices using TTC staining and ImageJ software (version 1.30, US National Institutes of Health, Bethesda, MD, USA) to investigate the changes in the cerebral infarction area in rats 6 hr after permanent ischemia (Figure 1c). After TTC staining, the cerebral infarction appeared as a white region in the blood‐supplying region of the right middle cerebral artery, adjacent to the surrounding red area with a relatively normal blood supply. At 6 hr after permanent MCAO, the proportion of the brain infarct area was significantly reduced in the leptin group compared with the vehicle group. Significant reductions in the proportion of the cerebral infarct area were observed in the third (*p* < 0.001) and second brain slices (*p* < 0.05) in the leptin group compared with the vehicle group (Figure 1d).

### 3.3 Leptin increased mitochondrial STAT3 phosphorylation in cerebral ischemia

**Figure 2:**
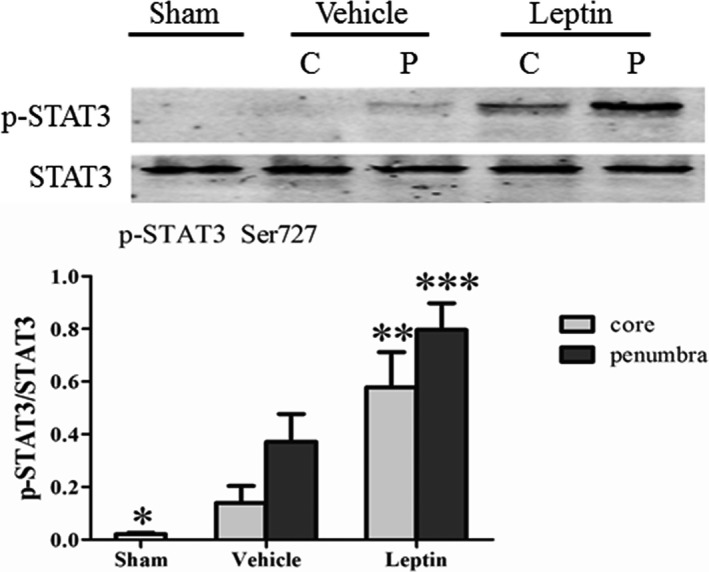
Leptin promotes STAT3 phosphorylation in the mitochondria after 6 hr of focal cerebral ischemia. Changes in STAT3 phosphorylation observed after focal cerebral ischemia were determined using a western blot analysis of phospho‐STAT3 (Ser727; p‐STAT3) and total STAT3 levels in mitochondria isolated from the ischemic core (C) and the penumbra (P) of rats treated with vehicle or leptin and sacrificed at 6 hr after MCAO. ^*^p < 0.001, compared with the vehicle (penumbra) and leptin groups (core and penumbra); ^**^p < 0.01, compared with the vehicle (core); ^***^p < 0.01, compared with the vehicle (penumbra) (*n* = 4 rats per experimental group)

We measured the levels of mitochondrial STAT3 phosphorylation (Ser727; p‐STAT3) and total STAT3 in the ischemic core and penumbra after 6 hr of ischemia using a western blot assay to investigate the effects of leptin on mitochondrial STAT3 phosphorylation during ischemia in rats (Figure 2). In addition, the purity of the isolated mitochondria was also assessed by western blotting (Appendix A1). The levels of phosphorylated STAT3 in mitochondria from the cerebral cortex revealed faintly detectable p‐STAT3 immunoreactivity in the sham group and in the ischemic core of the vehicle group. However, moderate mitochondrial p‐STAT3 levels were detected at in the penumbral region of the vehicle group. Mitochondrial STAT3 phosphorylation was further increased by the leptin treatment in rats at 6 hr after MCAO. Indeed, except for the ischemic core of the vehicle group, mitochondrial STAT3 phosphorylation was significantly increased in the vehicle and leptin groups compared to the sham group (*p* < 0.001). Moreover, higher levels of phosphorylated STAT3 were observed in the ischemic penumbra than in the core region (*p* < 0.05). The western blot analysis revealed a significantly higher level of mitochondrial STAT3 phosphorylation in the ischemic core (*p* < 0.01) and penumbra (*p* < 0.01) of the leptin group than in the vehicle group at 6 hr after MCAO (Figure 2).

### 3.4 Leptin reduced ROS production during cerebral ischemia

**Figure 3:**
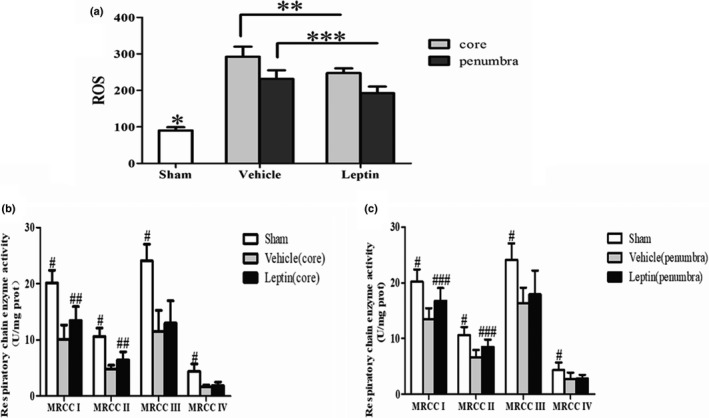
Leptin reduces ROS production (a) and slightly increases the activities of mitochondrial respiratory chain enzymes in the ischemic core (b) and penumbral (c) regions in rats at 6 hr after permanent MCAO. Total ROS levels in *SD* rats at 6 hr after MCAO were measured using a spectrophotometer. ^*^p < 0.001, compared with the vehicle and leptin groups (core and penumbra); ^**^p < 0.01, compared with the vehicle group (core); ^***^p < 0.01, compared with the vehicle group (penumbra) (*n* = 5 rats per experimental group). Mitochondrial respiratory chain enzymatic activities were quantified using a dual wavelength/single wavelength ultraviolet spectrophotometer. *^#^p* < 0.01, compared with the vehicle group (core and penumbra); *^##^p* < 0.05, compared with the vehicle group (core); *^###^p* < 0.05, compared with the vehicle group (penumbra) (*n* = 4 rats per experimental group)

Reactive oxygen species such as superoxide radicals, hydrogen peroxide, peroxides, and hydroxides are involved in oxidative stress processes. We examined the ischemia‐related changes in total ROS generation in the ischemic core and penumbral regions at 6 hr after permanent MCAO to determine whether leptin reduced the oxidative stress induced by ischemic injury in rats. The total ROS levels in the ischemic core and penumbral regions differed significantly compared with the sham group (*p* < 0.001). In addition, higher total ROS levels were observed in the core region than in the ischemic penumbra (*p* < 0.01), indicating that oxidative stress was more severe in the core region. Importantly, total ROS production was significantly reduced in the leptin group compared with the vehicle group (15.2% (*p* < 0.01) and 16.9% (*p* < 0.01) less than in the ischemic core and penumbral regions of the vehicle group, respectively) (Figure 3a).

### 3.5 Leptin slightly increased mitochondrial respiratory chain enzymatic activities during cerebral ischemia

During ischemia, mitochondrial respiratory chain enzymatic activities decrease to varying extents, which is an important factor in the generation of large amounts of ROS (Chen, Moghaddas, Hoppel, & Lesnefsky, 2008). We measured the enzymatic activities of the MRCCs (I‐IV) in the ischemic core and penumbra using an ultraviolet spectrophotometer to further evaluate the effects of leptin on mitochondrial oxidative stress at 6 hr after permanent MCAO in rats. As depicted in Figure 3b,c, the MRCC enzymatic activities were decreased after 6 hr of ischemia, and they were lower in the ischemic core than in the penumbra (*p* < 0.05). In addition, the enzymatic activities of MRCCs I and II were selectively increased in the core and penumbra of the leptin group compared with the vehicle group (*p* < 0.05). However, in the leptin group, the enzymatic activities of complexes III and IV were not affected in either the core or the penumbra.

## 4 DISCUSSION

In the current study, we investigated the association between leptin and mitochondrial oxidative stress after cerebral ischemia. We divided the infarct area into the ischemic core and penumbra to determine the effects of leptin on oxidative stress within these regions. Our results are consistent with previous studies revealing that leptin may exert its neuroprotective effects by reducing the infarct size, improving neurological function scores and reducing brain edema (Amantea et al., 2011; Zhang et al., 2013). In addition, as shown in the present study, leptin may affect the enzymatic activities of MRCCs I and II via the mitochondrial STAT3 signaling pathway to reduce ROS production in both the ischemic core and penumbra.

According to previous studies, leptin exerts a neuroprotective effect by inducing PI3K/Akt activation, which is related to energy metabolism (Zhang et al., 2013). Moreover, leptin may also relieve oxidative stress (Zhang et al., 2012), which may partially contribute to brain protection, but the exact mechanism remains to be elucidated. Our study provides the first evidence that the mitochondrial STAT3 signaling pathway may be involved in the antioxidant response of leptin. Ischemic damage increases ROS levels, and MRCCs I and III are the primary sources of ROS (Chen et al., 2008). Increased ROS production can disrupt a series of cell functions and ultimately lead to apoptosis (Simon, Haj‐Yehia, & Levi‐Schaffer, 2000). A steady increase in ROS formation has been observed in the ischemic cortex at 3 hr after permanent MCAO (Peters et al., 1998). In the present study, leptin reduced ROS formation during cerebral ischemia in rats. However, the effect of leptin on ROS formation remains controversial. Leptin has been shown to induce ROS formation, thereby contributing to cardiovascular remodeling (Ghantous et al., 2015) or anorexic behaviors (Palomba et al., 2015). In contrast, another study reported that a leptin treatment may improve bioenergetic efficiency by preventing ROS formation in breast cancer cells (Blanquer‐Rossello, Santandreu, Oliver, Roca, & Valle, 2015). Leptin may reduce ROS production by affecting mitochondrial function in cerebral ischemia.

The mitochondrial respiratory chain is damaged during cerebral ischemia (Almeida, Allen, Bates, & Clark, 1995; Tian et al., 2009). Based on the results from the present study, the enzymatic activities of the MRCCs decreased to different degrees after ischemic injury. Furthermore, leptin only protected respiratory chain complexes I and II from ischemic injury and had no significant effect on complexes III and IV. Moreover, the role of leptin in reducing ROS formation is not significantly associated with the protein levels of MRCCs in breast cancer cells (Blanquer‐Rossello et al., 2015). Thus, leptin slightly increases mitochondrial respiratory chain enzymatic activities to reduce ROS generation.

The neuroprotective effect of leptin is associated with an increase in STAT3 phosphorylation at Tyr705 in the nucleus or cytoplasm, particularly in the cortical penumbra, after permanent MCAO in rats (Amantea et al., 2011). In the current study, leptin promoted mitochondrial STAT3 phosphorylation at Ser727 in the cerebral ischemic core and similarly in the penumbra after permanent MCAO. An increase in the phosphorylation of mitochondrial STAT3 at Ser727 increases the enzymatic activities of MRCCs I and II, leading to the suppression of ROS production, the opening of mitochondrial permeability transition pores, and the release of cytochrome c during ischemia (Meier & Larner, 2014; Szczepanek, Lesnefsky, & Larner, 2012; Yang & Rincon, 2016). Generally, the number of STAT3 proteins in mitochondria is approximately one‐tenth the number in the cytoplasm, and following ischemic stimulation, STAT3 aggregates in mitochondria (Szczepanek, Chen, et al., 2012). NDUFA13 is a component of electron transfer chain complex I. Currently, the mechanism by which STAT3 is transported to the mitochondria remains unclear. NDUFA13, a molecular chaperone, has been shown to promote STAT3 translocation to the mitochondrial inner membrane. NDUFA13 probably changes the topology of mitochondrial STAT3 and promotes its integration into complex I (You et al., 2015). Recent studies have shown that STAT3 in mitochondria is not a necessary condition for maintaining normal mitochondrial function, but its existence is a necessary condition for maximizing the activity of mitochondrial electron transfer chain (ETC) complexes I and II. The relationship between these proteins may be mediated by protein—protein interactions or post‐transcriptional modifications (Gough et al., 2009; Szczepanek, Chen, et al., 2012). Interestingly, mitochondrial STAT3 has also recently been shown to regulate mitochondrial calcium homeostasis by affecting electron transport chains, the mitochondrial membrane potential, and the mitochondrial permeability transition pore, but little is known about the role of leptin (Yang et al., 2015; Yang & Rincon, 2016). Accordingly, the elevated levels of mitochondrial STAT3 Ser727 phosphorylation induced by leptin were associated with alterations in the enzymatic activities of MRCCs I and II in the present study, suggesting that the activation of mitochondrial STAT3 participates in the mechanism underlying the neuroprotective effects of leptin.

In summary, after permanent cerebral ischemia, the leptin treatment significantly promoted mitochondrial STAT3 phosphorylation in the ischemic core and penumbra, thus protecting respiratory chain I and II enzymatic activities from ischemic damage. These changes decreased ROS formation, reduced brain edema, improved neurological defects, reduced the infarct size, and eventually decreased experimental ischemic injury induced by permanent MCAO in rats. In general, leptin protects against the mitochondrial oxidative stress induced in the rat MCAO model, suggesting that it represents a potential therapeutic drug in the treatment of ischemic stroke.

## CONFLICTS OF INTEREST

The authors have no financial or other conflicts of interest related to this research and its publication to declare.

## APPENDIX 1

### Appendix A1

Mitochondrial (Mito) and cytosolic (Cyto) fractions were assessed using a Western blot analysis. Cytochrome c oxidase‐IV (COX‐IV) served as a mitochondrial marker and GAPDH served as a cytosolic marker to show the relative purity of mitochondrial fractions. Data are presented as mean percentages±standard deviations relative to the level of the Cyto group, n=4 rats per experimental group. COX‐IV levels in the Mito group were approximately 26 times higher than in the Cyto group. The GAPDH level in the Cyto group was approximately 13 times higher than in the Mito group. Western blot analysis revealed that the isolated mitochondria had high purity and low contamination levels.
