# Supplementary figures and images for: Leptin attenuates cerebral ischemic injury in rats by modulating the mitochondrial electron transport chain via the mitochondrial STAT3 pathway
Source: Brain Behav. 2019 Jan 10;9(2):e01200. doi: 10.1002/brb3.1200 (PMC6379515; doi:10.1002/brb3.1200)

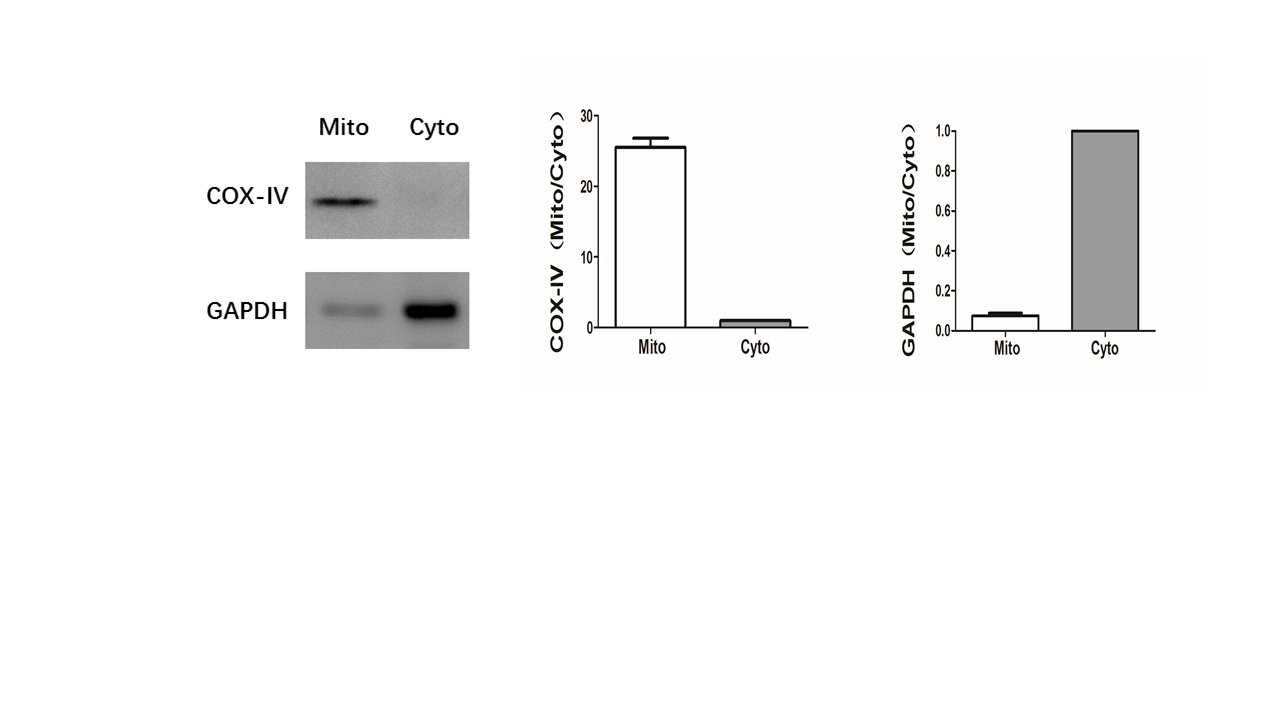

Supplement: Supplementary file 1 [file BRB3-9-e01200-s001.tif]
